# Supplementary material for: Structural and functional comparison of SARS-CoV-2-spike receptor binding domain produced in Pichia pastoris and mammalian cells
Source: Sci Rep. 2020 Dec 11;10:21779. doi: 10.1038/s41598-020-78711-6 (PMC7732851; doi:10.1038/s41598-020-78711-6)
Supplement: Supplementary file 1 — Supplementary Information. [file 41598_2020_78711_MOESM1_ESM.pdf]

Supplementary Material

Structural and Functional Comparison of SARS-CoV-2-Spike Receptor Binding Domain Produced in *Pichia pastoris* and Mammalian Cells

Argentinian AntiCovid Consortium†

† A comprehensive list of consortium members appears at the end of the paper. Correspondence and requests for materials should be addressed to the Argentinian AntiCovid Consortium (email: anticovid.arg@gmail.com).

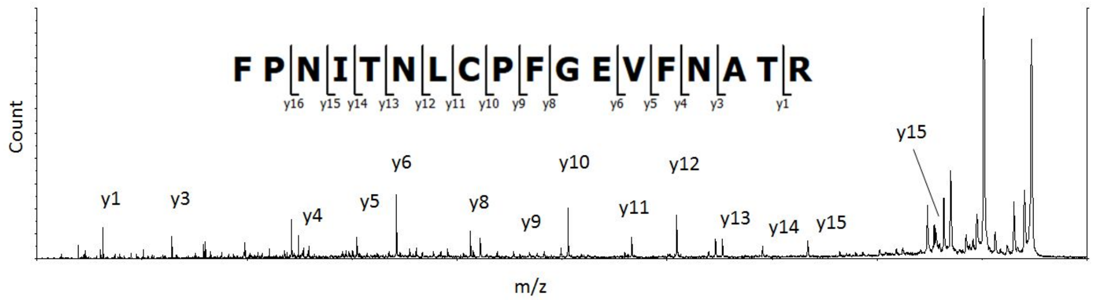

Figure S1. Signal assignment for the FPNITNLCPFGEVFNATR spectrum from *P. pastoris* RBD sample; in the inset, sequence assignment details.

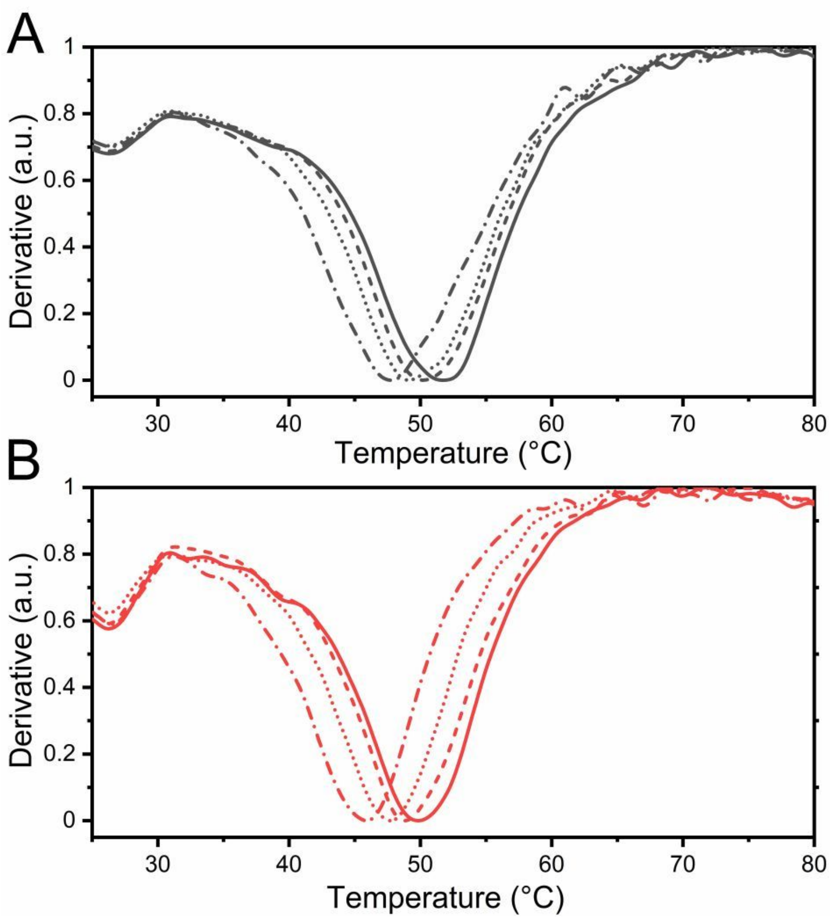

Figure S2. Derivative of temperature-induced Denaturation of RBD monitored by Sypro-Orange Fluorescence. Protein (5 μM) was prepared in buffer 50 mM sodium phosphate, pH 7.0, at different NaCl concentrations: 75 mM (solid line), 150 mM (dash line), 300mM (dot line) and 500mM (dash-dot line). (A) RBD obtained from HEK-293T and (B) RBD expressed on *P. pastoris.*

Author information:

Consortia:

Argentinian AntiCovid Consortium:

Claudia R. Arbeitman,[1,2,3] Gabriela Auge,[1,4] Matías Blaustein,[1,4] Luis Bredeston,[5,6] Enrique S. Corapi,[4] Patricio O. Craig,[7,8] Leandro A. Cossio,[9,22] Liliana Dain,[1,4,10] Cecilia D'Alessio,[1,4] Fernanda Elias,[11] Natalia B. Fernández,[1,4] Yamila B. Gándola,[4] Javier Gasulla,[1,4,12] Natalia Gorojovsky,[7,8] Gustavo E. Gudesblat,[1,4] María G. Herrera,[1,4] Lorena I. Ibañez,[13,14] Tommy Idrovo,[4] Matías Iglesias Rando,[5,8] Laura Kamenetzky,[4,15] Alejandro D. Nadra,[1,4] Diego G. Noseda[16], Carlos H. Paván,[17,18] María F. Pavan,[13,14] María F. Pignataro,[4] Ernesto Roman,[6,7] Lucas A. M. Ruberto,[19,20,21] Natalia Rubinstein,[1,4] Javier Santos,[1,4,7] Francisco Velazquez[4,8] and Alicia M. Zelada[9,22].

[1]Consejo Nacional de Investigaciones Científicas y Técnicas (CONICET). Godoy Cruz 2290 C1425FQB, Buenos Aires, Argentina.

[2]GIBIO-Universidad Tecnológica Nacional-Facultad Regional Buenos Aires. Medrano 951 C1179AAQ, Buenos Aires, Argentina.

[3]Theoretical Physics and Center of Interdisciplinary Nanostructure Science and Technology, Universität Kassel. Heinrich-Plett-Str. 40, 34132, Kassel, Germany.

[4]Universidad de Buenos Aires. Facultad de Ciencias Exactas y Naturales. Departamento de Fisiología y Biología Molecular y Celular. Instituto de Biociencias, Biotecnología y Biología Traslacional (iB3). Buenos Aires, Argentina.

[5]Universidad de Buenos Aires. Facultad de Farmacia y Bioquímica. Departamento de Química Biológica. Junín 965 C1113AAD. Buenos Aires, Argentina.

[6]CONICET-Universidad de Buenos Aires. Instituto de Química y Fisicoquímica Biológicas. (IQUIFIB). Buenos Aires, Argentina.

[7]Universidad de Buenos Aires. Facultad de Ciencias Exactas y Naturales. Departamento de Química Biológica. Buenos Aires, Argentina.

[8]CONICET-Universidad de Buenos Aires. Instituto de Química Biológica de la Facultad de Ciencias Exactas y Naturales (IQUIBICEN). Buenos Aires, Argentina.

[9]Universidad de Buenos Aires. Facultad de Ciencias Exactas y Naturales. Departamento de Fisiología y Biología Molecular y Celular. Laboratorio de Agrobiotecnología. Buenos Aires, Argentina.

[10]Centro Nacional de Genética Médica, Avda Las Heras 2670, 3er piso, C1425ASP, Buenos Aires, Argentina.

[11]Instituto de Ciencia y Tecnología Dr. César Milstein (Consejo Nacional de Investigaciones Científicas y Técnicas-Fundación Pablo Cassará). Saladillo 2468 C1440FFX, Buenos Aires, Argentina.

[12]Universidad Nacional de la Plata-CONICET. Centro de Investigaciones del Medio Ambiente (CIM). La Plata, Argentina.

[13]Consejo Nacional de Investigaciones Científicas y Técnicas (CONICET). Instituto de Ciencia y Tecnología Dr. César Milstein. Saladillo 2468, C1440FFX, Buenos Aires, Argentina.

[14]CONICET-Universidad de Buenos Aires. Instituto de Química Física de los Materiales, Medio Ambiente y Energía (INQUIMAE), Buenos Aires, Argentina.

[15]CONICET-Universidad de Buenos Aires. Facultad de Medicina. Instituto de Investigaciones en Microbiología y Parasitología (IMPaM). Buenos Aires, Argentina.

[16]Universidad Nacional de San Martín-CONICET. Instituto de Investigaciones Biotecnológicas (IIBio). San Martín, Buenos Aires, Argentina.

[17]Universidad de Buenos Aires. Facultad de Farmacia y Bioquímica. Buenos Aires, Argentina.

[18]CONICET-Universidad de Buenos Aires. Facultad de Medicina. LANAIS-PROEM. Instituto de Química y Fisicoquímica Biológicas. (IQUIFIB). Buenos Aires, Argentina.

[19]Universidad de Buenos Aires. Facultad de Farmacia y Bioquímica. Departamento de Microbiología, Inmunología, Biotecnología y Genética. Buenos Aires, Argentina.

[20]CONICET-Universidad de Buenos Aires. Facultad de Farmacia y Bioquímica. Instituto de Nanobiotecnología (NANOBIOTEC). Buenos Aires. Argentina.

[21]Instituto Antártico Argentino. Ministerio de Relaciones Exteriores y Culto. Buenos Aires, Argentina.

[22]CONICET-Universidad de Buenos Aires. Instituto de Biodiversidad y Biología Experimental y Aplicada (IBBEA). Buenos Aires, Argentina.

Contributions

The Argentinian AntiCovid Consortium performed the experimental work and drafted the manuscript. All authors (listed in alphabetical order) contributed equally to this work.
